# Multitasking as a Personal Choice of the Mode of Activity in Russian Children and Adolescents: Its Relationship to Experimental Multitasking and its Effectiveness

**DOI:** 10.11621/pir.2022.0208

**Published:** 2022-06-30

**Authors:** Galina U. Soldatova, Elena I. Rasskazova

**Affiliations:** a Lomonosov Moscow State University, Moscow, Russia; b Moscow Institute of Psychoanalysis, Moscow, Russia

**Keywords:** Subjective multitasking, personal choice, multitasking effectiveness, adolescents, children

## Abstract

**Background:**

A person’s ability to solve several tasks simultaneously, or within a limited amount of time, — *i.e.*, multitasking — is becoming more and more highly valued in society, despite experimental data in cognitive science about the low effectiveness of such activity. But, in the modern world, the term multitasking has become increasingly used in another sense — that is, a personal choice to perform several tasks simultaneously even if a person could do them consecutively.

**Objective:**

The aim of this study was to reveal the relationship between a personal preference for multitasking, its subjective effectiveness in children and adolescents, and their tendency for and efficacy of multitasking under experimental conditions.

**Design:**

One hundred and fifty-seven (157) schoolchildren of different ages participated in the study, which called for responding to four windows on a screen, including texts (SMS) and video images, and reporting on their subjective multitasking and its efficacy.

**Results:**

The majority of children and adolescents said (the older they were, the more likely) that sometimes, or often, they combine several tasks, and argued that their performance was effective.

**Conclusion:**

The subjective perspective on multitasking and its effectiveness was more likely to be related to multitasking by carrying out several tasks simultaneously, than switching between tasks, and was not related to actual effectiveness when undertaking a variety of activities within a limited time period. In the case of distractions (for instance, incoming messages while undertaking tasks), they might be related to a decrease in effectiveness.

## Introduction

The ability to solve several tasks simultaneously or within a limited time period is becoming more and more highly valued in different life spheres, despite experimental data in cognitive science about the low effectiveness of such practice (Kahneman, 1973; Pashler, 1994; Miller & Durst, 2015). Traditionally, the term multitasking suggests performing several tasks that often require the same level of cognitive load and are performed simultaneously, or within such a limited timeframe that a person has to make a choice between them.

However, in the modern world, the term multitasking has become increasingly used in another sense: that is, a personal choice to perform several tasks simultaneously even if a person could do them consecutively (Slocombe & Bluedorn, 1999; Poposki, Oswald, & Brou, 2009). The following reasons for such a choice can be provided. First, an analysis of the methods of multitasking according to their ecological validity (Ophir, Nass, & Wagner, 2009; Baumgartner, Lemmens, Weeda, & Huizinga, 2016) showed that often it comes from the personal choice to combine necessary but less desirable tasks with more pleasant ones (for instance, listening to music while mopping the floor). This is not classical multitasking, but a situation in which one activity is hierarchically auxiliary to the other one. In this case, it seems especially important to examine the functions of this “auxiliary” activity, a task which has not been performed to the best of our knowledge.

Second, the need to simultaneously carry out several tasks within a limited time period is becoming a more and more important social demand on individuals in many professions (Bühner, König, Pick, & Krumm, 2006). In other words, despite all the data showing that multitasking is ineffective, multitasking has developed into a modern value that is also becoming significant for adolescents. This theory is similar to that advanced in studies which emphasize the importance of controlling a shift from one task to another, and of changing one’s strategy (Oswald, Hambrick, & Jones, 2007).

Third, using information technology with its interactivity and combination of different contexts, Windows, and different digital devices causes people to refer to multitasking mode. But according to some data, the mere presence of an electronic device by your side can impair your performance (Ward, Duke, Gneezy, & Bos, 2017).

The results of a study on multitasking as carrying out within a certain time limit a number of tasks that do not require consecutiveness (for instance, to switch between tasks or to perform them simultaneously) allow us to suggest that we are talking about children and adolescents developing a specific socially accepted strategy of “saving time” (Soldatova & Rasskazova, 2020). At the ages of 11–13, this strategy starts to emerge in different and often ineffective attempts to save time that can often be seen as chaotic. By the ages of 14–17, the strategy of saving time becomes more consistent and more effective. For instance, while carrying out one of the tasks where you need to watch a video, older adolescents do not watch it completely but fast-forward it and occasionally stop as they think proper. This strategy proves to be more effective.

Our study found similar patterns related to the effectiveness of multitasking that were reflected in the principle of the memory development parallelogram defined by Leont’ev within Vygotsky’s Cultural-Historical psychology (Leont’ev, 2003). Using memory as an example, this principle reflects the pattern of higher mental functions development as a product of cultural-historical development. In general terms, it represents the transfer from direct forms of behavior to, at first, externally mediated actions with the help of stimuli-signs, and then to internally mediated forms that start to emerge within cultural development. In this case, the multitasking mode appeared to be one of the available functional uses of this principle, or as it sometimes called — rule. Therefore, we saw that, by the ages of 11–13, children can deal better with tasks of goal-oriented (the ones which were discussed in the instructions) multitasking, and that by the ages of 14–16, adolescents could tackle equally effectively both tasks requiring a goal-oriented activity (solving the tasks presented in the instructions) and those not requiring a goal-oriented activity (the instruction did not provide any information on those tasks. For instance, a sudden noise, music, etc.) (Soldatova & Rasskazova, 2020).

To understand multitasking as a personal choice, it must be pointed out that distractibility should not be included (Aagaard, 2019). From our point of view, the study results which showed that multitasking in one type of situations is not significantly related to multitasking in other types of situations (Redick, Shipstead, Meier, Montroy, Hicks, Unsworth, ... & Engle 2016) may not be connected not to the task’s content but to mixing automated and voluntary tasks. Furthermore, attempts to examine multitasking from the individual level — for instance, how, following the grounded theory, the multitasking phenomenon is presented in the adolescent mind (Lindstroem, 2020) — show that media multitasking is seen as distracting from another activity — that is, basically the equivalent of digital distractibility.

In this regard, attempts to develop an experimental method where you can simultaneously provide a set of tasks which comply with the requirements of ecological validity and include several diagnostic criteria, gain considerable significance. For instance, we suggested a new method for diagnosing media multitasking types among students. It was based on showing tasks in several windows on the screen and receiving questions via SMS while performing the tasks. The method also allowed us to compare simultaneous performance, postponing, and returning to tasks with performance effectiveness (Soldatova & Rasskazova, 2020).

The aim of this study was to reveal the relationship between the personal preference for multitasking and its subjective effectiveness in children and adolescents, and their tendency for and efficacy of multitasking in experimental conditions.

We hypothesized that:

1. Children and adolescents would report a high tendency for doing many tasks at the same moment. This tendency would be unrelated to gender, higher in older adolescents, and related to a higher subjective sense of the effectiveness of multitasking.
2. Subjective multitasking and subjective ideas about its efficacy would be weakly related or unrelated to experimentally induced media multitasking.

## Methods

### Participants

One hundred fifty-seven (157) schoolchildren took part in the study. Fifty-seven (57) were elementary school students age 7–11 years old (47.4% boys and 52.6% girls); 54 were adolescents age 11–13 years old who were in the 5th grade or (51.9% boys and 48.1% girls); and 46 were adolescents age 14–16 years old (54.3% boys and 45.7% girls).

### Materials

For evaluating multitasking as a respondent’s personal choice to combine different tasks and the subjective effectiveness of this choice, we asked the following questions:

1. “How often do you carry out several tasks simultaneously?” (The answer options were “I prefer to carry out only one task,” “Sometimes,” “Often,” “Always,” and “Not sure.”); and
2. “If you carry out several tasks simultaneously, you usually …” (The answer options were: “do not carry them out well,” “carry them out not so well,” “carry out some of them well and some of them badly.” “carry out all the task well,” and “Not sure”). Upon data processing, the option “Not sure” was considered as missing answer.

To evaluate media multitasking, an experimental complex was used which included a set of tasks typical for children and adolescents that had slightly different levels of difficulty depending on the children’s age. They were provided simultaneously in four different computer windows at the respondent’s home (messages were sent to respondents’ personal phones). The tasks were: 1) searching online a definition of an unknown word; 2) solving arithmetic and anagram tasks; 3) reading a text; 4) watching a short video; and 5) answering three questions sent by SMS every two minutes during the experiment.

While the youth performed the tasks, five musical segments were played in the background (croaking frogs, drums, guitar music, a chorus from a children’s song, and the sound of waves). After the children finished the tasks, an interviewer asked several questions about the tasks which the instructions called for (the message of the parable, word definition) and the tasks that were not discussed up front (*i.e.,* identifying the sound, recalling video details). The interviewer registered the fulfillment of every task, and the child’s strategy for answering the messages and watching the video. A video of the computer screen was also recorded.

### Procedure

Based on the experimental results, the following criteria for evaluating different aspects of multitasking and its effectiveness were used and then analyzed.

1. For evaluating **multitasking effectiveness**, there were two criteria: One was the effectiveness of performing the tasks requiring goal-oriented maintenance of the multitasking mode, and the other, the tasks which arose independent of the goal. The first subgroup had the tasks that were rotated with the others, but the respondents were warned that it was important to be sure to complete them: that is, tasks like solving arithmetic problems and anagrams, retelling a parable, or looking for the definition of the word turpentine. The second subgroup included the tasks where the respondents were asked questions without being warned beforehand: that is, questions about the sounds that they heard during their work and whether there were certain objects in the video. The scores for each scale were standardized for the whole sample and then averaged out.
2. **Multitasking as a shift between tasks,** including postponing and returning to the tasks, was graded by the number of shifts between the tasks: a respondent started a task, then postponed it to do some other tasks, and then returned to that task later. The lowest value of the number of shifts (“0”) meant that a student completed the entire task at the first attempt. Additionally, to understand the reason for switching, we calculated two types of time: absolute time (from the moment a student started the first task which he/she later postponed to when the student decided to postpone the task and do some other tasks); and relative time (the average time of carrying out all the postponed tasks, relative to the average time of solving all the tasks that were completed at the first attempt).
3. When possible, additional criteria for multitasking such as shifting tasks, the strategy for answering the questions received by SMS during the experiment, and the strategy for watching the video were included. Accordingly, the children and adolescents were divided into 1) those who performed the tasks consecutively (at the beginning, middle, and end of the experiment, they allocated a certain amount of time to answer all the messages at once); 2) those who switched between the tasks (they got distracted and answered the messages chaotically while performing a different task); and 3) those who could not answer all the messages.
  Significantly, these criteria do not allow us to clearly distinguish which strategy was used to answer the messages, and to evaluate its effectiveness (because we cannot evaluate the strategy of those who did not answer), and also to clearly divide switching between the tasks and simultaneous performance (in some cases students who answered chaotically could answer, for example, while watching the video). The strategy for watching the video was evaluated using a screen record and was divided into completely watched, not watched until the end, and fast-forwarded. We believed that not watching until the end, and especially fast-forwarding, might indicate attempts to optimize and speed up task completion and be connected to switching between tasks.
4. **Multitasking as a simultaneous task performance** was evaluated using the binary variable “Yes/No” in relation to the screen recording if a student performed tasks in other windows simultaneously with watching the video.

**Data analysis** was carried out using SPSS Statistics 23.0. Following the recommendations set out by Krichevets et al. (2019), we used both parametric and nonparametric methods to compare our results. While all the patterns we found were similar, below we represent the results of the parametric data processing.

## Results

### Multitasking as a personal choice: subjective evaluation of choosing multitasking and its effectiveness

**Figure 1.**
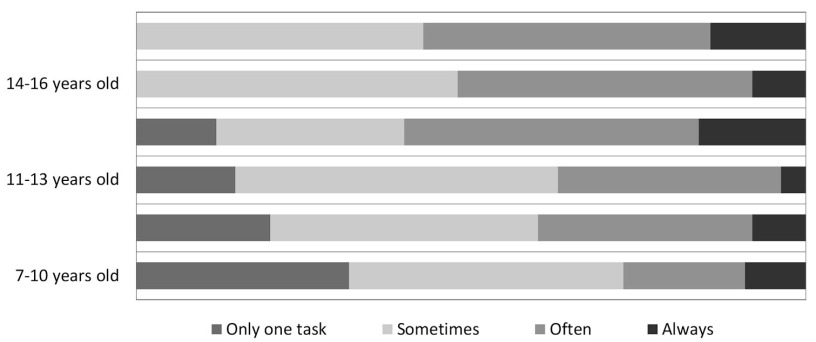
Frequency of performing several tasks simultaneously among boys and girls of different ages

Among the elementary school students, every fourth student preferred to do only one task at a time (25.5%); 40.4% preferred to do several tasks together at least some of the time; every fourth child (25.5%) often preferred doing several tasks simultaneously; and only one out of 12 always did several tasks at the same time (*Figure 1*). The older the child, the more likely he/she was to answer that they do several tasks simultaneously (χ^2^= 14.27, p<0.05, Cramer’s V = 0.22). By the ages of 14–16, no one said that they preferred to do only one task at a time. The absolute majority sometimes or often did several tasks. Interestingly, the percentage of those who said that they always did several tasks was consistently low among all ages; one out of 10 to 12 (8.5–10.9%) said that. No differences between boys and girls in subjective multitasking frequency were found overall or in any age group.

**Figure 2.**
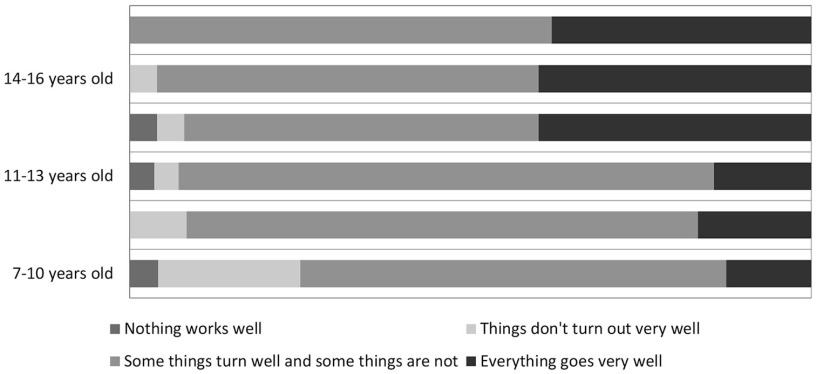
Subjective effectiveness of performing several tasks simultaneously among boys and girls of different ages

The majority of children and adolescents of all ages said that when they performed several tasks simultaneously, they could do some well and some not so well. However, on the whole the elementary school children were more skeptical about their success; the most confident in their abilities to do several tasks simultaneously were the adolescents’ ages 14–16 years old (*Figure 2*, χ^2^= 13.88, p < 0.05, Cramer’s V = 0.22). No differences between boys and girls in their subjective multitasking effectiveness were found overall or in any age group.

**Figure 3.**
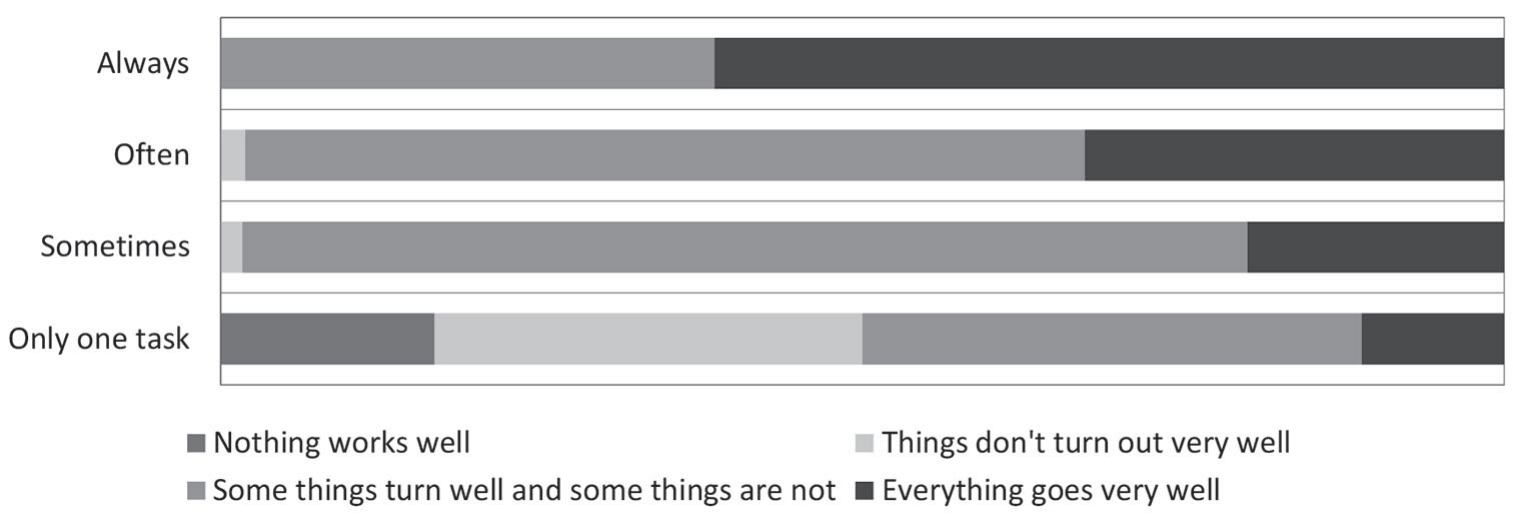
Comparing subjective frequency and effectiveness of performing several tasks simultaneously

Subjective frequency and the effectiveness of performing several tasks simultaneously are closely related to each other: the fact that combining tasks may be ineffective was almost exclusively admitted by the students who preferred to do only one task at a time (*Figure 3*, χ^2^= 63.31, p < 0.05, Cramer’s V = 0.38). The majority of children and adolescents combining tasks often or sometimes believed that they did some well and some not so well, while the majority of children and adolescents who always combined tasks believed that they did everything well.

### Subjective multitasking and task switching during the experiment

In general, the sample’s perception of their subjective multitasking frequency among all age groups was related to their strategy for answering SMS during the experiment (F = 3.19, p < 0.05, η^2^= 0.05). The children who had not answered all the messages said that they more often did several tasks simultaneously, compared to the children who had answered SMS in order, or when they were received (therefore postponing the other tasks).

Neither the frequency of the children and adolescents’ performance of several tasks simultaneously, nor their perception of the effectiveness of their performance, were related to how they performed the experimental tasks, their effectiveness in completing the tasks, or their strategy for watching the video. When we carried out a correlation analysis separately for each age group, the only correlation which reached the level of significance (p < 0.05) was that for adolescents 14–16 years old. There was a positive correlation between how often these adolescents performed several tasks simultaneously with the relative time within which they did the first task that they decided to postpone, in order to return to it later (r = 0.34, p < 0.05).

This correlation is explained by the fact that, among the older adolescents, the children who more often simultaneously performed several tasks at once, on average, at a trend level completed the tasks faster than the children who did several tasks simultaneously less often (r = –0.27, p < 0.08). And on average the children who more often simultaneously did several tasks at once made the decision to postpone a task after longer attempts to complete it; however, this effect was slightly below even the trend level (r = –0.23, p < 0.14). This relationship of subjective multitasking frequency and relative time before postponing the first task distinguished the adolescents age 14–16 years old from the adolescents age 11–13 years old (Z = 2.44, p < 0.05) and, at a trend level, from the elementary school students (Z = –1.94, p < 0.06) where the correlation was insignificantly negative.

Note that the adolescents who, according to the screen records, more often did the tasks simultaneously with watching the video, at a trend level, rated their abilities to effectively perform several tasks simultaneously most highly (t = –1.81, p < 0.08, η^2^= 0.02).

## Discussion

### Subjective multitasking among children and adolescents

According to their subjective evaluation, the majority of the children and adolescents, irrespective of sex, at least sometimes did several tasks simultaneously. Among the elementary school students, only one child out of four reported that they preferred to do only one task at a time. Closer to the age of older adolescents, none of the students chose this answer. In general, these data correspond with the results of our previous study where the criteria for evaluating multitasking differed from the criteria for its evaluation in this article (Soldatova, CHigar’kova, Drenyova, & Koshevaya, 2020). Furthermore, the children and adolescents tended to rate how effectively they perform the tasks highly; their ratings increased by the time of adolescence.

### Subjective multitasking and subjective evaluation of performance effectiveness

It is important that neither the frequency nor the subjective evaluation of doing several tasks simultaneously was related to how successful the children or adolescents were in completing the experimental tasks requiring switching attention. This result correlates with the data about the importance of considering which tasks and which areas of life we are talking about (Redick et al., 2016). In our opinion, the matter of simultaneously performing several tasks is more likely to characterize children and adolescents’ life strategy in this context in the following way: to what extent are they trying to do several tasks simultaneously while believing that their approach is effective.

However, the answers we received might not relate to the children’s real abilities to successfully accomplish multitasking. Moreover, subjective attempts to perform tasks simultaneously are more likely to relate (at a trend level) to the person’s actual strategy for performing tasks simultaneously, and, by older adolescence, to their strategy for performing tasks (at a trend level) faster and postponing only tasks that definitely take much more time, but not to the strategy of switching faster or more often between tasks while organizing the process of their performance.

On the contrary, in this experiment, the children who subjectively more often attempted to perform several tasks simultaneously were also more often unable to cope with the task of answering messages while doing other tasks, because they could not choose a single strategy for how to answer (consecutively or while performing the other tasks). This result is completely consistent with the data about heavy multitaskers presented in studies specifically based on subjective criteria of multitasking (Ophir, Nass, & Wagner, 2009). Thus, modern children often say that they combine several tasks and do them rather effectively. However, when it actually comes to tasks requiring attention, very few confident students really make an effort to do several tasks simultaneously (there were seven students in the sample), and they do not lead in effectiveness. And if it is necessary to choose when to react to unexpected distracting tasks, they are more likely to lose in effectiveness. The data allow us to suggest that, by older adolescence, children who subjectively more often do several tasks simultaneously develop the strategy of postponing tasks in the context of multitasking; they postpone tasks and resume them later only if those tasks take more time than the ones solved at the first approach.

## Conclusion

In general, in accordance with our data, the majority of children and adolescents said (the older they were, the more likely they were to say it) that sometimes or often they combined several tasks and asserted their effectiveness in doing so. However, this subjective evaluation of their multitasking and effectiveness was more likely to be related to multitasking as carrying out tasks simultaneously, rather than as switching between tasks, and was not related to their actual effectiveness when undertaking a variety of activities within a limited time period. And in the case of distracting tasks (for instance, SMS), the practice can be related to a decrease in effectiveness.

## Limitations

The major limitation of this study lay in its correlational design. All the tasks were presented to all the participants, thus allowing them to choose the order of the tasks and to either complete or postpone some of them. Although such a design is more ecologically valid than preassigned order of the tasks, it does not allow for detailed comparisons of switches between different tasks. Moreover, assessment of subjective multitasking was based on only two items. Although the interviewers controlled how children and adolescents understood these items, there could have been variations in what kinds of activities the participants considered to be multitasking. For example, there could have been differences related to age, parental behavior, and family rules. Further studies could compare different indicators of subjective multitasking and its efficacy.
